# Upper Semicontinuity of Pullback Attractors for the 3D Nonautonomous Benjamin-Bona-Mahony Equations

**DOI:** 10.1155/2014/853139

**Published:** 2014-03-26

**Authors:** Xinguang Yang, Xiaosong Wang, Juntao Li, Lingrui Zhang

**Affiliations:** ^1^College of Mathematics and Information Science, Henan Normal University, Xinxiang 453007, China; ^2^College of Information Science, Henan University of Technology, Zhengzhou 450001, China; ^3^College of Education and Teacher Development, Henan Normal University, Xinxiang 453007, China

## Abstract

We will study the upper semicontinuity of pullback attractors for the 3D nonautonomouss Benjamin-Bona-Mahony equations with external force perturbation terms. Under some regular assumptions, we can prove the pullback attractors *𝒜*_*ε*_(*t*) of equation $u_{t}-\Delta u_{t}-\nu \Delta u+\nabla \cdot \vec{F}(u)=ɛg(x,t)$ , *x* ∈ Ω, converge to the global attractor *𝒜* of the above-mentioned equation with *ε* = 0 for any *t* ∈ ℝ.

## 1. Introduction

In this paper, we will consider the upper semicontinuity of pullback attractors for the following 3D Benjamin-Bona-Mahony equation:

(1) $$\begin{array}{c} u_{t}-\Delta u_{t}-\nu \Delta u+\nabla \cdot \vec{F}\left(u\right)=ɛg\left(x,t\right),\;x\in \Omega , \end{array}$$

(2) $$\begin{array}{c} {u(t,x)|}_{\partial \Omega }=0, \end{array}$$

(3) $$\begin{array}{c} u\left(\tau ,x\right)=u_{\tau }\left(x\right),\;\tau \in \mathbb{R}. \end{array}$$

Here *Ω* ⊂ ℝ^3^ is a bounded domain with sufficiently smooth boundary ∂*Ω*; *u*(*t*, *x*) = (*u*_1_(*t*, *x*), *u*_2_(*t*, *x*), *u*_3_(*t*, *x*)) is the velocity vector field; *ν* > 0 is the kinematic viscosity; $\vec{F}$ is a nonlinear vector function; *ɛ* ≥ 0 is a small nonnegative parameter; the external force *g*(*x*, *t*) is locally square integrable in time for (*x*, *t*) ∈ *Ω* × ℝ, that is, for any *t* ∈ ℝ, *g*(*x*, *t*) ∈ *L*_loc⁡_^2^(ℝ; *H*), where *H* = (*L* ^2^(*Ω*))^3^, *V* = (*H*_0_^1^(*Ω*))^3^, and (·, ·) and ||·|| are the inner product and norm of *H*, respectively.

The Benjamin-Bona-Mahony (BBM) equation is a well-known model in physical applications which incorporates dispersive effects for long waves in shallow water that was introduced by Benjamin et al. [1] as an improvement of the Korteweg-de Vries equation (KdV equation) for modeling long waves of small amplitude in two dimensions. Contrasting with the KdV equation, the BBM equation is unstable in its high wave number components. Further, while the KdV equation has an infinite number of integrals of motion, the BBM equation only has three. Both KdV and BBM equations cover cases of surface waves of long wavelength in liquids, acoustic-gravity waves in compressible fluid, hydromagnetic waves in cold plasma, and acoustic waves in harmonic crystals.

For the well-posedness of global solutions for BBM equation, we can refer to [2–7]. For the long-time behavior, such as the existence of global attractor and its structure and the dimension of the attractors, we will discuss the known results in details.

Biler [8] investigated the long-time behavior of 2D generalized BBM equation

(4) $$\begin{array}{c} u_{t}-\Delta u_{t}=\left(b,\nabla u\right)+u^{p}\left(a,\nabla u\right) \end{array}$$

in ℝ^2^, *t* ∈ ℝ. Here *b* ≠ 0, *a* ∈ ℝ^2^, and *p* ≥ 3 is an integer. The author proved the supremum norms of the solutions with small initial data decay to zero like *t* ^−2/3^ as *t* tends to infinity.

By energy equation and weak continuous method, Wang [9] and Wang and Yang [10] investigated the finite-dimensional behavior of solutions and derived the global weak attractor and the strong attractors for BBM equation:

(5) $$\begin{array}{c} u_{t}-u_{xxt}-\nu u_{xx}+{\left(f\left(u\right)\right)}_{x}=g\left(x\right) \end{array}$$

with period boundary value condition in *H*_per_^2^(*Ω*) and *H*_per_^1^(*Ω*), respectively. Moreover, Wang et al. [11] got the existence of global attractor for the above BBM equation defined in a three-dimensional channel; the asymptotic compactness of the solution operator is obtained by the uniform estimates on the tails of solutions.

By the decomposition of the semigroup, Wang [12] studied the regularity of attractors for the BBM equation

(6) $$\begin{array}{c} u_{t}-u_{xxt}-\nu u_{xx}+u_{x}+uu_{x}=g\left(x\right). \end{array}$$

He proved that the global attractor is smooth if the forcing term is smooth. In addition, Wang [13] also obtained the approximate inertial manifolds to the global attractors for the generalized BBM equations.

Wang [14] considered the stochastic BBM equations on unbounded domains

(7) $$\begin{array}{c} du-d\left(\Delta u\right)-\nu \Delta udt+\nabla \cdot \vec{F(u)}dt=gdt+hdw \end{array}$$

and concluded the existence of random attractor in *H*_0_^1^ under certain assumptions, here *w* is the two-sided real-valued Wiener process on a probability space. He also proved the random attractor is invariant and attracts every pulled-back tempered random set under the forward flow. The asymptotic compactness of the random dynamical system is established by a tail-estimates method, which shows that the solutions are uniformly asymptotically small when space and time variables approach infinity.

Stanislavova et al. [15] first provided a sufficient condition to verify the asymptotic compactness of an evolution equation defined in an unbounded domain, which involves the Littlewood-Paley projection operators, then they proved the existence of an attractor for the Benjamin-Bona-Mahony equation in the phase space *H* ^1^(ℝ^3^) by showing the solutions are point dissipative and asymptotic compact

(8) $$\begin{array}{c} u_{t}-\Delta u_{t}-\nu \Delta u+\mathrm{div}\left(f\left(u\right)\right)=g \end{array}$$

for *g* ∈ *L* ^2^(ℝ^3^) and *f*(*u*) = *u* + (1/2)*u* ^2^. Stanislavova [16] investigated the existence of global attractors of (8) in two dimension.

By the method of orthogonal decomposition, Zhu [17, 18] obtained the asymptotic attractor, global attractor, and its Hausdorff dimension of the damped BBM equations with periodic boundary conditions in homogeneous periodic space ${\dot{H}}_{\text{per}}^{1}(\Omega )$

(9) $$\begin{array}{c} u_{t}-\delta u_{xxt}-\nu u_{xx}+uu_{x}=f\left(x\right) \end{array}$$

which overcome difficulty coming from the precision of approximate inertial manifolds. Zhu and Mu [19] deduced the exponential decay estimates of solutions for time-delayed BBM equations.

J. Park and S. Park [20] studied the pullback attractors for the nonautonomous BBM equations in unbounded domains

(10) $$\begin{array}{c} u_{t}-\Delta u_{t}-\nu \Delta u+\nabla \cdot \vec{F\left(u\right)}\;=g\left(x,t\right), \end{array}$$

by weak continuous method and some priori estimates in *H*_0_^1^(*Ω*). Qin et al. [21] derived the existence of pullback attractor of (10) in *H*_0_^2^(*Ω*) by weak continuous method. Zhao et al. [22] investigated the convergence of corresponding uniform attractors between averaging BBM and state BBM equations.

Moreover, Çelebi et al. [23] deduced the existence of attractors with a finite fractal dimension and the existence of the exponential attractor for the corresponding asymptotically compact semigroup for the periodic initial-boundary value problem of a generalized BBM equation. Chueshov et al. [24] studied the regularity of global attractor for a generalized BBM equation.

For the upper semicontinuity of corresponding attractors between autonomous and perturb nonautonomous systems, we can refer to Bao [25], Hale and Raugel [26], Carvalho et al. [27], Caraballo and Langa [28], Caraballo et al. [29], Fitzgibbon et al. [30], Kloeden [31], Miyamoto [32], Wang and Qin [33], Younsi [34], Wang [35], and Zhou [36].

To our knowledge, there are less results on the upper semicontinuity of pullback attractors for the 3D nonautonomous BBM equations with the nonautonomous perturbation; we will pay attention to this issue in the sequel.

This paper is organized as following. In Section 2, we will recall some fundamental theory of pullback attractors for nonautonomous dynamical systems and give a method to verify the upper semicontinuity of pullback attractors. In Section 3, the upper semicontinuity of pullback attractors for the problems (1)–(3) will be proved.

## 2. Pullback Attractors of Nonautonomous Dynamical Systems

In this section, we will consider the relationship between pullback attractors *𝒜*_*ɛ*_ = {*A*_*ɛ*_(*t*)}_*t*∈ℝ_ for the perturbed nonautonomous system with *ɛ* > 0 and global attractor *𝒜* for the unperturbed autonomous system with *ɛ* = 0 of the following equation:

(11) $$\begin{array}{c} \frac{\partial u}{\partial t}={𝔸}_{f}u\left(x,t\right)+ɛf\left(x,t\right). \end{array}$$

If the global attractor is unique, then the global attractor is the pullback attractor when *ɛ* = 0.

Let *X* be a Banach space with norm ||·||_*X*_. The Hausdorff semidistance dist⁡_*X*_(*B*_1_, *B*_2_) in *X* between *B*_1_⊆*X* and *B*_2_⊆*X* is defined by

(12) $$\begin{array}{c} {\mathrm{dist}}_{X}\left(B_{1},B_{2}\right)=\underset{x\in B_{1}}{\sup}\;\underset{y\in B_{2}}{\inf}d_{X}\left(x,y\right)\;\text{for}\;B_{1},B_{2}\subset X, \end{array}$$

where *d*_*X*_(*x*, *y*) denotes the distance between two points *x* and *y*.

For an autonomous system, *S*(*t*) : *X* → *X* (*t* ∈ ℝ) is a *C*_0_-semigroup defined on *X*. If the global attractor *𝒜* for *S*(*t*) exists, then it has the following properties: (1) *𝒜* is an invariant, compact set; (2) *𝒜* attracts every bounded sets in *X*, that is, lim⁡_*t*→+*∞*_⁡dist⁡(*S*(*t*)*B*, *𝒜*) = 0 for all bounded subsets *B* ⊂ *X*.

For a nonautonomous system, the two-parameter mapping class {*U*(*t*, *τ*)}_*t*≥*τ*_ is said to be a process in *X* if

(13) $$\begin{array}{c} U\left(t,s\right)U\left(s,\tau \right)=U\left(t,\tau \right),\;\forall t\geq s\geq \tau ,\;\;\tau \in \mathbb{R},\\ U\left(\tau ,\tau \right)=Id,\left(\text{identity}\;\text{operator}\;\text{in}\;X\right),\;\forall \tau \in \mathbb{R}. \end{array}$$

Moreover, throughout the paper, we always assume that the process *U*(·, ·) is continuous in *X*.

Now we will recall some definitions and framework on the existence theory of pullback attractors.

**Definition 1**

A family of compact sets *𝒜* = {*A*(*t*)}_*t*∈ℝ_ is said to be a pullback attractor for the continuous process {*U*(·, ·)} if it satisfies the following:

- *𝒜* is invariant for all *t* ≥ *τ*.
- *𝒜* is pullback attracting, that is, lim⁡_*τ*→+*∞*_⁡dist⁡(*U*(*t*, *t* − *τ*)*B*, *A*(*t*)) = 0 for all bounded subsets *B* ⊂ *X*.

**Definition 2**

The family of subsets *ℬ* = {*B*(*t*)}_*t*∈ℝ_ is said to be pullback absorbing for the process *U*(·, ·), if for every *t* ∈ ℝ and all bounded subsets *B* ⊂ *X*, there exists a time *T*(*t*, *B*) > 0, such that

(14) $$\begin{array}{c} U\left(t,t-\tau \right)B\subset B\left(t\right)\;\forall \tau \geq T\left(t,B\right). \end{array}$$

**Definition 3**

Let *ℬ* = {*B*(*t*)}_*t*∈ℝ_ be a family of subsets in *X*. A process *U*(·, ·) is said to be pullback *ℬ*-asymptotically compact in *X* if for all *t* ∈ ℝ, any sequences *τ*_*n*_ → *∞* and *x*_*n*_ ∈ *B*(*t* − *τ*_*n*_); the sequence {*U*(*t*, *t* − *τ*_*n*_)*x*_*n*_} is precompact in *X*.

**Theorem 4**

Let the family of sets *ℬ* = {*B*(*t*)}_*t*∈ℝ_ be pullback absorbing set for the process *U*(·, ·) and *U*(·, ·) is pullback *ℬ*-asymptotically compact in *X*. Then, the family *𝒜* = {*A*(*t*)}_*t*∈ℝ_ that is defined by *A*(*t*) = Λ(*ℬ*, *t*) is a pullback attractor for *U*(·, ·) in *X* for the process {*U*(·, ·)}, where

(15) $$\begin{array}{c} \Lambda \left(ℬ,t\right)=\underset{s\geq 0}{⋂}\;\bar{\underset{\tau \geq s}{⋃}U\left(t,t-\tau \right)B\left(t-\tau \right)}\;for\;each\;t\in \mathbb{R}. \end{array}$$

In the following, we will characterize the pullback *ℬ*-asymptotic compactness in terms of the noncompact measure.

**Definition 5**

Let *B* ⊂ *X*, *ℬ* = {*B*(*t*)}_*t*∈ℝ_ be a family of sets in *X*. A process *U*(·, ·) is said to be pullback *ℬ*-*κ* contracting, if for any *t* ∈ ℝ, *ɛ* > 0, there exists a time *T*_*ℬ*_(*t*, *ɛ*) > 0, such that

(16) $$\begin{array}{c} \kappa \left(U\left(t,t-\tau \right)B\left(t-\tau \right)\right)\leq ɛ\;\forall \tau \geq T_{ℬ}\left(t,ɛ\right). \end{array}$$

Here *κ*(*B*) is the Kuratowski noncompact measure defined as

(17) κ(B)=inf⁡{δ>0 ∣ B  admits  a  finite  cover         by  sets  of  diameter<δ}.

**Lemma 6**

Let *ℬ* = {*B*(*t*)}_*t*∈ℝ_, $\hat{ℬ}={\left\{\hat{B}(t)\right\}}_{t\in \mathbb{R}}$ be two families of sets in *X* and satisfy that for any *t* ∈ ℝ, there exists a time $T_{ℬ,\hat{ℬ}}(t)>0$ , such that

(18) $$\begin{array}{c} U\left(t,t-\tau \right)B\left(t-\tau \right)\subset \hat{B}\left(t\right)\;\forall \tau \geq T_{ℬ,\hat{ℬ}}\left(t\right). \end{array}$$

Then *U*(·, ·) is pullback *ℬ*-asymptotically compact, if it is pullback $\hat{ℬ}$ -*κ* contracting.

**Proof**

See, for example, Wang and Qin [33].

**Theorem 7**

Assume that the assumptions in Lemma 6 hold. If the process *U*(·, ·) is pullback $\hat{ℬ}$ -*κ* contracting and the family of sets *ℬ* = {*B*(*t*)}_*t*∈ℝ_ is pullback absorbing for *U*(·, ·), then the process *U*(·, ·) possesses a pullback attractor.

**Proof**

See, for example, Wang  and  Qin [33].

**Theorem 8**

Let *ℬ* = {*B*(*t*)}_*t*∈ℝ_ be a family of sets in *X*. Suppose *U*(·, ·) = *U*_1_(·, ·) + *U*_2_(·, ·) : ℝ × ℝ × *X* → *X* satisfies

(i) for any *t* ∈ ℝ,
(19) $$\begin{array}{c} {||U_{1}\left(t,t-\tau \right)x_{t-\tau }||}_{X}\leq \Phi \left(t,\tau \right)\;\forall x_{t-\tau }\in B\left(t-\tau \right),\;\;\tau >0, \end{array}$$
 where Φ(·, ·) : ℝ × ℝ → ℝ^+^ satisfies lim⁡_*τ*→+*∞*_⁡Φ(*t*, *τ*) = 0 for each *t* ∈ ℝ;
(ii) for any *t* ∈ ℝ and *T* ≥ 0, ⋃_0≤*τ*≤*T*_ *U*_2_(*t*, *t* − *τ*)*B*(*t* − *τ*) is bounded and *U*_2_(*t*, *t* − *τ*)*B*(*t* − *τ*) is precompact in *X* for any *τ* > 0.

Then the process *U*(·, ·) is pullback *ℬ*-*κ* contracting in *X*.

**Proof**

See, for example, Wang and Qin [33].

We now perturb the nonautonomous term with a small parameter *ɛ* ∈ (0, *ε*_0_]; thus we obtain a nonautonomous dynamical system driven by the process *U*_*ɛ*_(·, ·).

For each *t* ∈ ℝ,  *τ* ∈ ℝ, and *x* ∈ *X*, we have

(20) $$\begin{array}{c} \left(H_{1}\right)\;\underset{ɛ\to 0}{\lim}d_{X}\left(U_{ɛ}\left(t,t-\tau \right)x,S\left(t\right)x\right)=0, \end{array}$$

uniformly on bounded sets of *X*.

**Theorem 9 (Caraballo et al. [28, 29])**

Assume that (*H*_1_) holds, and for any *ɛ* ∈ (0, *ε*_0_], there exist pullback attractors *𝒜*_*ɛ*_ = {*A*_*ɛ*_(*t*)}_*t*∈ℝ_ for all *ɛ* > 0. If there exists a compact set *K* ⊂ *X*, such that

(21) $$\begin{array}{c} \left(H_{2}\right)\;\underset{ɛ\to 0}{\lim}dis_{X}\left(A_{ɛ}\left(t\right),K\right)=0\;for\;any\;t\in \mathbb{R}. \end{array}$$

Then *𝒜*_*ɛ*_ and *𝒜* have the upper semicontinuity, that is,

(22) $$\begin{array}{c} \underset{ɛ\to 0}{\lim}dis_{X}\left(A_{ɛ}\left(t\right),𝒜\right)=0\;for\;any\;t\in \mathbb{R}. \end{array}$$

In order to apply Theorem 9 to obtain the upper semicontinuity of pullback attractors *𝒜*_*ɛ*_ and global attractor *𝒜*, we now present a technique to verify (*H*_2_) for the process generated by the nonautonomous dissipative system.

**Lemma 10**

Assume that the family *ℬ* = {*B*(*t*)}_*t*∈ℝ_ is pullback absorbing for *U*(·, ·), and for each *ɛ* ∈ (0, *ε*_0_], *𝒦*_*ɛ*_ = {*K*_*ɛ*_(*t*)}_*t*∈ℝ_ is a family of compact sets in *X*. Suppose *U*_*ɛ*_(·, ·) = *U*_1,*ɛ*_(·, ·) + *U*_2,*ɛ*_(·, ·) : ℝ × ℝ × *X* → *X* satisfies

(i) for any *t* ∈ ℝ and any *ɛ* ∈ (0, *ε*_0_],
(23) $$\begin{array}{c} {||U_{1,ɛ}\left(t,t-\tau \right)x_{t-\tau }||}_{X}\leq \Phi \left(t,\tau \right)\;\forall x_{t-\tau }\in B\left(t-\tau \right),\;\;\tau >0, \end{array}$$
 where Φ(·, ·) : ℝ × ℝ → ℝ^+^ satisfies lim⁡_*τ*→+*∞*_⁡Φ(*t*, *τ*) = 0 for each *t* ∈ ℝ;
(ii) for any *t* ∈ ℝ and any *T* ≥ 0, ∪_0≤*τ*≤*T*_ *U*_2,*ɛ*_(*t*, *t* − *τ*)*B*(*t* − *τ*) is bounded, and for any *t* ∈ ℝ, there exists a time *T*_*ℬ*_(*t*) > 0, which is independent of *ɛ*, such that
(24) $$\begin{array}{c} U_{2,ɛ}\left(t,t-\tau \right)B\left(t-\tau \right)\subset K_{ɛ}\left(t\right)\;\forall \tau \geq T_{ℬ}\left(t\right),\;\;ɛ\in \left(0,\varepsilon_{0}\right] \end{array}$$
 and there exists a compact set *K* ⊂ *X*, such that
(25) $$\begin{array}{c} \left(H_{2}^{\prime }\right)\;\underset{ɛ\to 0}{\lim}dist_{X}\left(K_{ɛ}\left(t\right),K\right)=0,\;for\;any\;t\in \mathbb{R}. \end{array}$$
 Then for each *ɛ* ∈ (0, *ε*_0_], there exists a pullback attractor *𝒜*_*ɛ*_ = {*A*_*ɛ*_(*t*)}_*t*∈ℝ_ and (*H*_2_) holds.

**Proof**

See, for example, Wang and Qin [33].

## 3. Upper Semicontinuity of Pullback Attractors

In this section, firstly, we recall some notations about the functional spaces which will be used later to discuss the regularity of pullback attracting set.

The operator *A* is denoted by *A* = −Δ with domain *D*(*A*) = *H* ^2^(*Ω*)⋂*H*_0_^1^(*Ω*) and *λ* is the first eigenvalue of *A*; we consider the family of Hilbert spaces

(26) $$\begin{array}{c} {ℋ}^{\alpha }=D\left(A^{\alpha /2}\right),\;\alpha \in \mathbb{R} \end{array}$$

generated by the Laplacian operator with the Dirichlet boundary value conditions equipped with the standard inner product and norm

(27) $$\begin{array}{c} {\left(\cdot ,\cdot \right)}_{{ℋ}^{\alpha }}=\left(A^{\alpha /2}\cdot ,A^{\alpha /2}\cdot \right),\;\;{||\cdot ||}_{{ℋ}^{\alpha }}=||A^{\alpha /2}\cdot || \end{array}$$

respectively, then we have *D*(*A* ^*s*/2^)↪*D*(*A* ^*r*/2^) for any *s* > *r* and the continuous embedding

(28) $$\begin{array}{c} {ℋ}^{s}\equiv D\left(A^{s/2}\right)↪{\left(L^{6/\left(3-2s\right)}\left(\Omega \right)\right)}^{3} \end{array}$$

for all *s* ∈ [0, 3/2), *ℋ* ^2^ = *H* ^2^(*Ω*)⋂*H*_0_^1^(*Ω*).

Then, applying the Helmholtz-Leray projector *𝒫* to the systems (1)–(3), we obtain the following problem which is equivalent to the original problems (1)–(3)

(29) $$\begin{array}{c} u_{t}+\nu Au+Au_{t}+B\left(u\right)=ɛf\left(x,t\right),\;\left(x,t\right)\in \Omega \times \left[\tau ,\infty \right),\\ u\left(x,t\right)=0,\;\left(x,t\right)\in \partial \Omega \times \left[\tau ,\infty \right),\\ u\left(\tau ,x\right)=u_{\tau }\left(x\right),\;x\in \Omega . \end{array}$$

Here *A* = −*𝒫*Δ, $B(u)=𝒫(\nabla \cdot \vec{F}(u))$ , and *f*(*x*, *t*) = *𝒫g*(*x*, *t*).

Assume that *u*_*τ*_ ∈ *H*_0_^1^(*Ω*), the external force *g* ∈ *L*_loc⁡_^2^(ℝ, *H*). Also we assume that there exist constants *β* > 0, 0 ≤ *α* < *σ*/2, and *σ* = 2*ν*/((2/*λ*) + 2), such that

(30) $$\begin{array}{c} {||g(t)||}^{2}\leq \beta e^{\alpha |t|}, \end{array}$$

which implies that

(31) $$\begin{array}{c} \overset{t}{\underset{-\infty }{\int }}e^{\sigma s}{||g(s)||}^{2}ds<+\infty ,\;\forall t\in \mathbb{R},\\ \overset{\tau }{\underset{-\infty }{\int }}\left(\overset{t}{\underset{-\infty }{\int }}e^{\sigma s/2}{||g(s)||}^{2}ds\right)dt<+\infty ,\;\forall \tau \in \mathbb{R}. \end{array}$$

Moreover, we assume that

(32) $$\begin{array}{c} \underset{k\to \infty }{\lim}\overset{\tau }{\underset{-\infty }{\int }}\int_{|x_{3}|\geq k}e^{\sigma t}{\left|g\left(x,t\right)\right|}^{2}dx\;dt=0,\;\forall \tau \in \mathbb{R}. \end{array}$$

From (31), we can easily derive that the term *f*(*x*, *t*) is locally square integrable in time; that is, *f*(*x*, *t*) ∈ *L*_loc⁡_^2^(ℝ, *H*) and satisfies

(33) $$\begin{array}{c} \overset{t}{\underset{-\infty }{\int }}e^{\eta s}{||f(x,t)||}_{H}^{2}ds<+\infty \end{array}$$

for 0 < *η* ≤ min⁡{*λν*, *ν*, *ν*/((2/*λ*) + 2)} and any *t* ∈ ℝ.

For the nonlinear vector function $\begin{array}{c} \vec{F}(s) \end{array}=(F_{1}(s),F_{2}(s),F_{3}(s))\;(s\in \mathbb{R})$ , we denote

(34) $$\begin{array}{c} f_{i}\left(s\right)=F_{i}^{\prime }\left(s\right),\;\;{ℱ}_{i}\left(s\right)=\overset{s}{\underset{0}{\int }}F_{i}\left(r\right)dr, \end{array}$$

where

(35) f→(s) =(f1(s),f2(s),f3(s)),ℱ→(s) =(ℱ1(s),ℱ2(s),ℱ3(s)).

Assume that *F*_*i*_ (*i* = 1,2, 3) are smooth functions satisfying

(36) Fi(0)=0,  |Fi(s)|≤C1|s|+C2|s|2,C1(1+σ2|s|)≤|fi(s)|≤C2(1+σ2|s|),|ℱi(s)|≤C1|s|2+C2|s|3,

for all *s* ∈ ℝ, where *C*_1_, *C*_2_, and *σ* are positive constants.

At last, we will state the main result and the proof of this paper as the following.

**Theorem 11**

Assume that (30)–(36) hold, and *u*_*τ*_ ∈ *V*, then the pullback attractors *𝒜*_*ɛ*_ = {*𝒜*_*ɛ*_(*t*)}_*t*∈ℝ_ for (29) (which is equivalent to (1)) with *ɛ* > 0 and the global attractor *𝒜* for (29) with *ɛ* = 0 satisfy

(37) $$\begin{array}{c} \underset{ɛ\to 0^{+}}{\lim}dis_{V}\left({𝒜}_{ɛ}\left(t\right),𝒜\right)=0\;for\;any\;t\in \mathbb{R}. \end{array}$$

The Hausdorff semidistance *dist*(·, ·) is defined on the Banach space *V*.

In order to apply Theorem 9 and Lemma 10 to prove Theorem 11, we will introduce the existence of global attractor for autonomous system (1) with *ɛ* = 0 and pullback attractors for nonautonomous system (1) with *ɛ* > 0 in the following lemmas.

**Lemma 12**

Assume that (34)–(36) hold, and *u*_*τ*_ ∈ *V*, then the semigroup *S*(*t*) (*t* ∈ ℝ) generated by problem (29) (or problems (1)–(3)) with *ɛ* = 0 possesses a global attractor *𝒜* in *V*.

**Proof**

Using similar technique as in [9–11, 17, 18], we only need to consider the Dirichlet boundary value condition instead of the periodic boundary value condition in these papers which investigated the existence of global attractors. This means that we can obtain our lemma easily, here we omit the details.

**Lemma 13**

Assume that (30)–(36) hold, and *u*_*τ*_ ∈ *V*, then problem (29) possesses a unique global solution *u* ^*ɛ*^(*x*, *t*) (*ɛ* ≥ 0) satisfying

(38) $$\begin{array}{c} u^{ɛ}\left(x,t\right)\in C\left(\left[\tau ,+\infty \right),V\right)\cap L^{\infty }\left(0,+\infty ;V\right),\\ u_{t}\in L^{2}\left(0,T;V\right). \end{array}$$

Moreover, the process {*U*_*ɛ*_(*t*, *τ*)} generated by the global solutions possess pullback attractors *𝒜*_*ɛ*_ for all *ɛ* ≥ 0 in *V*.

**Proof**

See, for example, [20].

Now we decompose the solution *u* ^*ɛ*^(*t*) = *U*_*ɛ*_(*t*, *τ*)*u*_*τ*_ of (29) with initial data *u*_*τ*_ ∈ *V* as

(39) $$\begin{array}{c} u^{ɛ}=U_{ɛ}\left(t,\tau \right)u_{\tau }=U_{1,ɛ}\left(t,\tau \right)u_{\tau }+U_{2,ɛ}\left(t,\tau \right)u_{\tau }, \end{array}$$

where

(40) $$\begin{array}{c} U_{1,ɛ}\left(t,\tau \right)u_{\tau }=v\left(t\right),\\ U_{2,ɛ}\left(t,\tau \right)u_{\tau }=w\left(t\right) \end{array}$$

solve the following problems:

(41) $$\begin{array}{c} v_{t}+Av_{t}+\nu Av+B\left(v\right)=0,\;\text{in}\;\left(x,t\right)\in \Omega \times \left[\tau ,\infty \right),\\ v\left(x,t\right)=0,\;\mathrm{on}\;\partial \Omega \times \left[\tau ,\infty \right),\\ v\left(\tau ,x\right)=v_{\tau }\left(x\right),\;x\in \Omega , \end{array}$$

(42) wt+Awt+νAw =−B(u)+B(v)+ɛf(x,t), in (x,t)∈Ω×[τ,∞),w(x,t)=0, on⁡ ∂Ω×[τ,∞),w(τ,x)=0, x∈Ω,

respectively.

**Lemma 14**

Suppose that (34)–(36) hold. For any bounded set *B* ⊂ *V* and *t* ∈ ℝ, there exists a time *T*(*B*, *t*) > 0, such that

(43) ||Uɛ(t,t−τ)ut−τ||2≤Rɛ(t)∀τ≥T(B,t),  all  ut−τ∈B,

where *R*_*ɛ*_(*t*) = *Cɛe* ^−*ηt*^∫_−*∞*_^*t*^ *e* ^*ηs*^||*f*(*s*)||_*H*_^2^ *ds*, and *C* is a positive constant independent of *B*, *t*, *τ*.

**Proof**

We choose *σ* = 2*ν*/((2/*λ*) + 2), *R*_*σ*_ = {*r* : *R* → (0, +*∞*) | lim⁡_*t*→−*∞*_ *e* ^*σt*^ *r* ^2^(*t*) = 0} and denote by *𝒟*_*σ*_ the class of families $\hat{D}=\{D(t):t\in R\}\subset 𝒟(H)$ such that $D(t)\subset \bar{B}(0,r_{\hat{D}}(t))$ for some $r_{\hat{D}}$ , where $\bar{B}(0,r_{\hat{D}}(t))$ denotes the closed ball in *V* centered at zero with radius $r_{\hat{D}}(t)$ .

Let *t* ∈ ℝ,  *τ* ∈ ℝ, and *u*_*τ*_ ∈ *V* be fixed, and denote

(44) u(r)=u(r;t−τ,u0)=U(r−t+τ,t−τ,u0) for  r≥t−τ.

Since *u* ∈ *C*((*τ*, *T*); *V*), then for all *u* ∈ *V*, we derive that

(45) ddt(eσt||u(t)||2+eσt||∇u(t)||2)  +2νeσt||∇u(t)||2 =−2eσt(∇F→(u),∇u)  +σ(eσt||u(t)||2+eσt||∇u||2)  +2eσt(ɛf(t),u(t)) =σ(eσt||u(t)||2+eσt||∇u||2)  +2eσt(ɛf(t),u(t)) ≤σ(1λ+1)eσt||∇u||2+σλeσt||∇u||2  +ɛσeσt||f(t)||H2 ≤σ(2λ+1)eσt||∇u(t)||2+ɛσeσt||f(t)||H2 ≤2νeσt||∇u(t)||2+ɛσeσt||f(t)||H2,

that is,

(46) $$\begin{array}{c} \frac{d}{dt}\left(e^{\sigma t}{||u(t)||}^{2}+e^{\sigma t}{||\nabla u(t)||}^{2}\right)\leq \frac{ɛ}{\sigma }e^{\sigma t}{||f(t)||}_{H}^{2}, \end{array}$$

which gives

(47) ||u(t)||2+||∇u(t)||2 ≤e−σ(t−τ)(||uτ||2+||∇uτ||2)  +ɛσ∫τte−σ(t−ξ)||f(ξ)||H2dξ

for all *τ* ∈ ℝ.

Let $\hat{D}\in {𝒟}_{\sigma }$ be given, choosing appropriate parameter *σ*, we easily get

(48) $$\begin{array}{c} {||U(t,\tau ,u_{\tau })||}_{V}^{2}\leq e^{-\sigma (t-\tau )}r_{\hat{D}}^{2}+\frac{ɛ}{\sigma }\overset{t}{\underset{-\infty }{\int }}e^{-\sigma (t-\xi )}{||f(\xi )||}_{H}^{2}d\xi , \end{array}$$

for all *u*_*τ*_ ∈ *D*(*τ*),  *t* ≥ *τ*.

Setting $e^{-\sigma (t-\tau )}r_{\hat{D}}^{2}\leq (ɛ/\sigma )\int_{-\infty }^{t}e^{-\sigma (t-\xi )}{||f(\xi )||}_{H}^{2}d\xi$ , then we denote *R*_*ɛ*_(*t*) the nonnegative number given for each *t* ∈ ℝ by

(49) $$\begin{array}{c} {\left(R_{ɛ}\left(t\right)\right)}^{2}=\frac{2ɛ}{\sigma }\overset{t}{\underset{-\infty }{\int }}e^{-\eta (t-\xi )}{||f(\xi )||}_{H}^{2}d\xi , \end{array}$$

and consider the family ${\hat{B}}_{ɛ}$ of closed balls in *V* defined by

(50) $$\begin{array}{c} B_{ɛ}\left(t\right)=\left\{v\in V\;|\;{||v||}_{V}\leq 2R_{ɛ}\left(t\right)\right\}. \end{array}$$

It is straightforward to check that ${\hat{B}}_{ɛ}\in {𝒟}_{\sigma }$ and hence ${\hat{B}}_{\sigma }$ is the *𝒟*_*σ*_-pullback absorbing for the process {*U*(*t*, *τ*, *u*_*τ*_)}.

Setting

(51) $$\begin{array}{c} B_{ɛ}=\left\{u\in V\;|\;{||u||}_{V}\leq R_{ɛ}\left(t\right)\right\}, \end{array}$$

then we can check that family *ℬ*_*ɛ*_ = {*B*_*ɛ*_(*t*)}_*t*∈ℝ_ is pullback absorbing in *V* easily. Moreover,

(52) $$\begin{array}{c} \underset{t\to -\infty }{\lim}e^{\eta t}R_{ɛ}\left(t\right)=0\;\text{for}\;\text{any}\;ɛ>0. \end{array}$$

**Lemma 15**

Let *R*_*ɛ*_(*t*), *B*_*ɛ*_(*t*) be given as above. For any *t* ∈ ℝ, the solution *v*(*t*) = *U*_1,*ɛ*_(*t*, *t* − *τ*)*u*(*t* − *τ*) of (41) satisfies

(53) $$\begin{array}{c} {||U_{1,ɛ}(t,t-\tau )u_{t-\tau }||}_{V}^{2}\leq e^{-\eta \tau }R_{ɛ}\left(t-\tau \right), \end{array}$$

for all *τ* ≥ 0 and *u*_*t*−*τ*_ ∈ *D*_*ɛ*_(*t* − *τ*).

**Proof**

Multiplying equation in (41) with *v* and integrating over *Ω*, we derive

(54) $$\begin{array}{c} \frac{1}{2}\frac{d}{dt}\left({||v(t)||}^{2}+{||\nabla v(t)||}^{2}\right)+\nu {||\nabla v||}^{2}\leq 0. \end{array}$$

Here we use the property of operator *B*(·) and *ℱ*_*i*_(0) = 0 as

(55) ∫Ω(∇·F→(u))udx=−∫ΩF→(u)·∇udx=−∫Ω∇·ℱ→(u)dx=−∫∂Ωℱ→(u)·n→dx=0,

where $\vec{n}$ is the outer unit normal vector.

Using Poincaré's inequality, it follows

(56) $$\begin{array}{c} \frac{d}{dt}\left({||v(t)||}^{2}+{||\nabla v(t)||}^{2}\right)+\eta \left({||v||}^{2}+{||\nabla v||}^{2}\right)\leq 0, \end{array}$$

where we set 0 < *η* ≤ min⁡{*λ*_1_ *ν*, *ν*}.

Integrating (56) from *t* − *τ* to *t*, we get

(57) ||U1,ɛ(t,t−τ)ut−τ||V2≤||v(t)||2+||∇v(t)||2≤(||vt−τ||2+||∇vt−τ||2)e−η(t−τ)≤e−ητRɛ(t−τ)

for all *t* ≥ *τ*, which completes our proof.

**Lemma 16**

Let *ℬ*_*ɛ*_(*t*) = {*B*_*ɛ*_(*t*)}_*t*∈ℝ_ be given by (51) and (52). For any *t* ∈ ℝ, there exist a time *T*_*ɛ*_(*t*, *ℬ*) > 0 and a function *I*_*ɛ*_(*t*) > 0, such that the solution *U*_2,*ɛ*_(*t*, *τ*)*u*_*τ*_ = *w*(*t*) of (42) satisfies

(58) $$\begin{array}{c} {||U_{2,ɛ}(t,t-\tau )u_{t-\tau }||}_{V}^{2}\leq I_{ɛ}\left(t\right), \end{array}$$

for all *τ* ≥ *T*_*ɛ*_(*t*, *ℬ*) and any *u*_*t*−*τ*_ ∈ *B*_*ɛ*_(*t* − *τ*).

**Proof**

Taking the inner product of equation in (42) with *A* ^*σ*^ *w*(*t*) in *H*, we derive

(59) 12ddt(||Aσ/2w(t)||2+||A(σ+1)/2w(t)||2)  +ν∫Ω|A(σ+1)/2w(t)|2dx =−〈B(u),Aσw〉+〈B(v),Aσw〉  +ɛ〈f(t,x),Aσw〉.

By Poincaré's inequality, Lemma 15, (51), and (34)–(36), we obtain

(60) −〈B(u),Aσw〉+〈B(v),Aσw〉 ≤|〈∇·(F→(v)−F→(u)),Aσw〉| ≤|(sup⁡i=1,2,3(|F→i′(v)|+|F→i′u|)   ×(∇v−∇u),Aσw)| =|(sup⁡i=1,2,3(|F→i′(v)|+|F→i′u|)∇w,Aσw)| ≤||sup⁡i=1,2,3(|F→i′(v)|+|F→i′u|)||||A(σ+(1/2))/2w|| ≤C(ɛ)λ||sup⁡i=1,2,3(|F→i′(v)|+|F→i′u|)||2  +νλ||A(σ+(1/2))/2w||2 ≤C(ɛ)λ||sup⁡i=1,2,3(|F→i′(v)|+|F→i′u|)||2  +ν||A(σ+1)/2w||2 ≤Cλ(2+σ2||u||2+σ2||v||2)  +ν||A(σ+1)/2w||2 ≤C(2+σ2Rɛ(t)+σ2e−ητRɛ(t−τ))  +ν||A(σ+1)/2w||2,〈f(t,x),Aσw〉≤1λ||A(σ+1)/2w(t)||2+λ||f(t)||H2.

Hence according to (59)–(60) and (31), we have

(61) ddt(||Aσ/2w(t)||2+||A(σ+1)/2w(t)||2) ≤C(1+ɛ||A(σ+1)/2w(t)||2+ɛ||f(t)||H2),

where the constant *C* depends on ||*u*_*t*−*τ*_||_*V*_^2^, *σ*, and the first eigenvalue *λ* of the operator *A*.

Integrating (61) from *t* − *τ* to *t*, we conclude that

(62) ||Aσ/2w(t)||2+||A(σ+1)/2w(t)||2 ≤CeCt∫t−τt(1+ε2||f(x,s)||2)e−Csds =Iɛ(t)

for all *t* > *τ*. This completes the proof of desiring lemma.

**Lemma 17**

For any *t* ∈ ℝ, any *τ* > 0, if *u*_0_ varies in bounded sets, then the solution *u*_*ɛ*_(*t*) = *U*_*ɛ*_(*t*, *t* − *τ*)*u*_0_ of problem (1) converges to the solution *u*(*t*) = *S*(*t*)*u*_0_ of the unperturbed problem (1) with *ɛ* = 0 uniformly in *V* as *ɛ* → 0^+^, which means

(63) $$\begin{array}{c} \underset{ɛ\to 0^{+}}{\lim}\;\underset{u_{0}\in B}{\sup}{||u_{ɛ}(t)-u(t)||}_{V}=0, \end{array}$$

where *B* is a bounded subset in *V*.

**Proof**

Denote

(64) $$\begin{array}{c} y^{ɛ}\left(t\right)=u_{ɛ}\left(t\right)-u\left(t\right), \end{array}$$

then we can verify that *y* ^*ɛ*^(*t*) satisfies

(65) ytϵ+Aytɛ+νAyɛ=−B(uɛ)+B(u)+ɛf(x,t),

(66) $$\begin{array}{c} {y_{ɛ}|}_{\partial \Omega }=0, \end{array}$$

(67) $$\begin{array}{c} {y_{ɛ}|}_{t=\tau }={\left(u_{ɛ}\right)}_{\tau }-u_{\tau }. \end{array}$$

Multiplying (65) by*y* ^*ɛ*^(*t*), using (34)–(36) and noting the boundary value condition (66), we have

(68) 12ddt(||yɛ||2+||∇yɛ||2)+ν||∇yɛ||2 =〈B(u),yɛ〉−〈B(uɛ),yɛ〉+〈ɛf,yɛ〉 ≤|〈B(uɛ)−B(u),yɛ)〉|+〈ɛf,yɛ〉 ≤|〈∇·(F→(uɛ)−F→(u)),yɛ〉|  +ε24ν||f(t)||H2+λν2||yɛ||2 =|〈F→(uɛ)−F→(u),∇yɛ〉|  +ε24λν||f(t)||H2+λν2||yɛ||2.

Using (34)–(36) and the Sobolev compact embedding theorem *V*↪*L* ^6^↪*L* ^4^↪*L* ^2^, we get

(69) |〈F→(uɛ)−F→(u),∇yɛ〉| ≤|(C1|uɛ|+C2|uɛ|2+C1|u|+C2|u|2,∇yɛ)| ≤Cν(||uɛ||2+||uɛ||L42+||u||2+||u||L42)  +ν2||∇yɛ||2 ≤Cλν(||uɛ||V2+||u||V2)+ν2||∇yɛ||2.

Hence

(70) 12ddt(||yɛ||2+||∇yɛ||2)+ν||∇yɛ||2 ≤Cλν(||uɛ||V2+||u||V2)+ν2||∇yɛ||2  +ε24λν||f(t)||H2+ν2||∇yɛ||2,

that is,

(71) ddt(||yɛ||2+||∇yɛ||2)≤Cλν(||uɛ||V2+||u||V2) +ε24λν||f(t)||H2.

Using Lemmas 13, 14, 15, 16, and (31), noting that *η* = min⁡{*λν*, *ν*, *ν*/((1/*λ*) + 2)}, we know

(72) uɛ, u∈C([τ,+∞),V),∫t−τt(||uɛ(s)||V2+||u(s)||V2)ds ≤Cɛ∫t−τte−ηt∫−∞teηs||f(s)||H2ds dt  +Cɛ∫t−τte−ηt∫−∞t−τeηs||f(s)||H2ds dt ≤Cɛ∬−∞teηs||f(s)||H2ds dt  +Cɛ∬−∞teηs||f(s)||H2ds dt≤Cɛ.

Applying the Gronwall inequality to (71) and noting that *f* ∈ *L*_loc⁡_^2^(ℝ, *H*), using Lemmas 14, 15, and 16, we conclude

(73) ||yɛ||V2≤C(||yɛ||2+||∇yɛ||2)≤Cɛ[∫t−τt(||uɛ(s)||V2+||u(s)||V2)ds   +∫t−τt||f(s)||H2ds]≤Cɛ[∫t−τte−ηt∫−∞teηs||f(s)||H2ds dt   +∫t−τte−ηt∫−∞t−τeηs||f(s)||H2ds dt] +Cε2[∫t−τt||f(s)||H2ds]≤C′ɛ⟶0

as *ɛ* → 0^+^, which implies (63).

**Proof of Theorem 11**

Since the embedding *D*(*A* ^*s*/2^)↪(*L* ^6/(3−2*s*)^(*Ω*))^3^ is compact, combining Lemmas 13–17 with Theorem 9 and Lemma 10, we can obtain Theorem 11 easily.

## References

[1] Benjamin TB, Bona JL, Mahony JJ (1972). Model equations for long waves in nonlinear dispersive systems. *Philosophical Transactions of the Royal Society A*.

[2] Arrieta JM, Carvalho AN, Rodríguez-Bernal A (1999). Perturbation of the diffusion and upper semicontinuity of attractors. *Applied Mathematics Letters*.

[3] Avrin J, Goldstein JA (1985). Global existence for the Benjamin-Bona-Mahony equation in arbitrary dimensions. *Nonlinear Analysis: Theory, Methods & Applications*.

[4] Caldas CSQ, Limaco J, Barreto RK (2007). About the Benjamin-Bona-Mahony equation in domains with moving boundary. *Trends in Applied and Computational Mathematics*.

[5] Guo BL (1987). Initial-boundary value problem for one class of systems of multidimensional inhomogeneous GBBM equations. *Chinese Annals of Mathematics. Series B*.

[6] Hărăguş M (2008). Stability of periodic waves for the generalized BBM equation. *Revue Roumaine de Mathématiques Pures et Appliquées*.

[7] Medeiros LA, Menzala GP (1977). Existence and uniqueness for periodic solutions of the Benjamin-Bona-Mahony equation. *SIAM Journal on Mathematical Analysis*.

[8] Biler P (1992). Long time behaviour of solutions of the generalized Benjamin-Bona-Mahony equation in two-space dimensions. *Differential and Integral Equations*.

[9] Wang B (1997). Strong attractors for the Benjamin-Bona-Mahony equation. *Applied Mathematics Letters*.

[10] Wang B, Yang W (1997). Finite-dimensional behaviour for the Benjamin-Bona-Mahony equation. *Journal of Physics A: Mathematical and General*.

[11] Wang B, Fussner DW, Bi C (2007). Existence of global attractors for the Benjamin-Bona-Mahony equation in unbounded domains. *Journal of Physics A: Mathematical and Theoretical*.

[12] Wang B (1998). Regularity of attractors for the Benjamin-Bona-Mahony equation. *Journal of Physics A: Mathematical and General*.

[13] Wang B (1997). Attractors and approximate inertial manifolds for the generalized Benjamin-Bona-Mahony equation. *Mathematical Methods in the Applied Sciences*.

[14] Wang B (2009). Random attractors for the stochastic Benjamin-Bona-Mahony equation on unbounded domains. *Journal of Differential Equations*.

[15] Stanislavova M, Stefanov A, Wang B (2005). Asymptotic smoothing and attractors for the generalized Benjamin-Bona-Mahony equation on ℝ^3^. *Journal of Differential Equations*.

[16] Stanislavova M (2005). On the global attractor for the damped Benjamin-Bona-Mahony equation. *Discrete and Continuous Dynamical Systems. Series A*.

[17] Zhu C (2007). Global attractor for the damped Benjamin-Bona-Mahony equations on ℝ^1^. *Applicable Analysis*.

[18] Zhu C (2008). Asymptotic attractors of Benjamin-Bona-Mahony equations. *European Journal of Pure and Applied Mathematics*.

[19] Zhu C, Mu C (2008). Exponential decay estimates for time-delayed Benjamin-Bona-Mahony equations. *Applicable Analysis*.

[20] Park JY, Park SH (2011). Pullback attractors for the non-autonomous Benjamin-Bona-Mahony equation in unbounded domains. *Science China Mathematics*.

[21] Qin Y, Yang X, Liu X (2012). Pullback attractors for the non-autonomous Benjamin-Bona-Mahony equations in *H*
^2^. *Acta Mathematica Scientia. Series B*.

[22] Zhao M, Yang X, Zhang L (2013). Averaging of the 3*D* non-autonomous Benjamin-Bona-Mahony equation with singularly oscillating forces. *Boundary Value Problems*.

[23] Çelebi AO, Kalantarov VK, Polat M (1999). Attractors for the generalized Benjamin-Bona-Mahony equation. *Journal of Differential Equations*.

[24] Chueshov I, Polat M, Siegmund S (2004). Gevrey regularity of global attractor for generalized Benjamin-Bona-Mahony equation. *Matematicheskaya Fizika, Analiz, Geometriya*.

[25] Bao TQ (2012). Existence and upper semi-continuity of uniform attractors for nonautonomous reaction diffusion equations on ℝ^
*n*
^. *Electronic Journal of Differential Equations*.

[26] Hale JK, Raugel G (1988). Upper semicontinuity of the attractor for a singularly perturbed hyperbolic equation. *Journal of Differential Equations*.

[27] Carvalho AN, Rodrigues HM, Dlotko T (1998). Upper semicontinuity of attractors and synchronization. *Journal of Mathematical Analysis and Applications*.

[28] Caraballo T, Langa JA (2003). On the upper semicontinuity of cocycle attractors for non-autonomous and random dynamical systems. *Dynamics of Continuous, Discrete and Impulsive Systems Series A*.

[29] Caraballo T, Langa JA, Robinson JC (1998). Upper semicontinuity of attractors for small random perturbations of dynamical systems. *Communications in Partial Differential Equations*.

[30] Fitzgibbon WE, Parrott ME, You Y (1996). Finite dimensionality and upper semicontinuity of the global attractor of singularly perturbed Hodgkin-Huxley systems. *Journal of Differential Equations*.

[31] Kloeden PE (2006). Upper semi continuity of attractors of delay differential equations in the delay. *Bulletin of the Australian Mathematical Society*.

[32] Miyamoto Y (2006). Upper semicontinuity of the global attractor for the Gierer-Meinhardt model. *Journal of Differential Equations*.

[33] Wang Y, Qin Y (2010). Upper semicontinuity of pullback attractors for nonclassical diffusion equations. *Journal of Mathematical Physics*.

[34] Younsi A (2014). Upper semicontinuous trajectory attractors for 3D hyperviscous flow. *Mediterranean Journal of Mathematics*.

[35] Wang B (2009). Upper semi-continuity of random attractors for non-compact random dynamical systems. *Electronic Journal of Differential Equations*.

[36] Zhou S (2012). Upper-semicontinuity of attractors for random lattice systems perturbed by small white noises. *Nonlinear Analysis: Theory, Methods & Applications*.

